# Translating qualitative data into intervention content using the Theoretical Domains Framework and stakeholder co-design: a worked example from a study of cervical screening attendance in older women

**DOI:** 10.1186/s12913-022-07926-2

**Published:** 2022-05-06

**Authors:** Alison Bravington, Hong Chen, Judith Dyson, Lesley Jones, Christopher Dalgliesh, Amée Bryan, Julietta Patnick, Una Macleod

**Affiliations:** 1 Hull York Medical School, University of Hull, Kingston-Upon-Hull, UK; 2 Warwick Medical School, University of Warwick, Coventry, UK; 3 Centre for Social Care, Health and Related Research, Birmingham City University, Birmingham, UK; 4 Durham University, Durham, UK; 5 Nuffield Department of Population Health, University of Oxford, Oxford, UK

**Keywords:** Cervical screening, Qualitative, Behaviour change, Theoretical domains framework, Stakeholder involvement, Intervention development

## Abstract

**Background:**

Previous screening interventions have demonstrated a series of features related to social determinants which have increased uptake in targeted populations, including the assessment of health beliefs and barriers to screening attendance as part of intervention development. Many studies cite the use of theory to identify methods of behaviour change, but fail to describe in detail how theoretical constructs are transformed into intervention content. The aim of this study was to use data from a qualitative exploration of cervical screening in women over 50 in the UK as the basis of intervention co-design with stakeholders using behavioural change frameworks. We describe the identification of behavioural mechanisms from qualitative data, and how these were used to develop content for a service-user leaflet and a video animation for practitioner training. The interventions aimed to encourage sustained commitment to cervical screening among women over 50, and to increase sensitivity to age-related problems in screening among primary care practitioners.

**Methods:**

Secondary coding of a qualitative data set to extract barriers and facilitators of cervical screening attendance. Barrier and facilitator statements were categorised using the Theoretical Domains Framework (TDF) to identify relevant behaviour change techniques (BCTs). Key TDF domains and associated BCTs were presented in stakeholder focus groups to guide the design of intervention content and mode of delivery.

**Results:**

Behavioural determinants relating to attendance clustered under three domains: beliefs about consequences, emotion and social influences, which mapped to three BCTs respectively: (1) persuasive communication/information provision; (2) stress management; (3) role modelling and encouragement. Service-user stakeholders translated these into three pragmatic intervention components: (i) addressing unanswered questions, (ii) problem-solving practitioner challenges and (iii) peer group communication. Based on (ii), practitioner stakeholders developed a call to action in three areas – clinical networking, history-taking, and flexibility in screening processes. APEASE informed modes of delivery (a service-user leaflet and a cartoon animation for practitioners).

**Conclusion:**

The application of the TDF to qualitative data can provide an auditable protocol for the translation of qualitative data into intervention content.

**Supplementary Information:**

The online version contains supplementary material available at 10.1186/s12913-022-07926-2.

## Background

Cancer of the cervix is one of the most preventable forms of the disease: pre-cancerous cells can be identified using a screening test and treated before they develop into cancer. Public cervical screening programmes are provided in many countries, but do not generally reach target participation rates [1]. Reviews of interventions to encourage screening uptake demonstrate that cervical screening programmes face different challenges to breast and colorectal screening [2, 3]. Cancer screening is targeted by age and gender: in England, women aged 50 to 70 are invited for breast screening, men and women aged 60 to 74 for colorectal cancer screening and women aged 25 to 64 for cervical screening. Cervical screening is stratified further, transitioning from 3-yearly to 5-yearly screening from the age of 50.

Cervical screening also differs from breast and colorectal screening in other ways. Screening the cervix is an invasive procedure, requiring a sample from inside an intimate area of the body. Having this procedure carried out by a GP or practice nurse can cause embarrassment or distress [4, 5]. Health beliefs surrounding cervical cancer can also affect attendance – for example, stigma and perceptions of risk arising from the association of cervical cancer with promiscuity [6–8]. Research into barriers that keep women from attending for screening suggests that a multiplicity of demographic and cultural factors also contribute to decision-making [9, 10], in addition to health knowledge and structural issues such as the costs associated with taking time off work or travelling to appointments [5, 11].

In 2019-20, a preliminary test was introduced for human papillomavirus (HPV), a common, symptomless infection which can be contracted from a single sexual contact and is the main causal factor in the development of cervical cancer. Prior to this test becoming standard in the UK, all screening samples were subject to cytology (examining cells from the cervix for pre-cancerous changes); under current protocols, only those which are positive for a high-risk strain of HPV are now taken forward. Vaccination to protect against HPV was introduced for girls aged 12-13 in the UK in 2008, with the eldest girls to benefit now aged 30-31. The vaccine is not routinely given to older women as it offers less protection and is less cost-effective [12], leaving them at greater risk. Home testing for HPV is currently being trialled in the UK [13]; if this approach is successful, women over 50 will need encouragement to engage with home testing. Where a HPV test is positive, they will subsequently need to attend their GP surgery for a cervical screening test.

Among the demographic factors, age is now playing a key role in the challenges facing cervical screening programmes. In the UK, a quarter of women aged 50 to 64 do not attend free screening offered by the National Health Service, and rates for attendance drop further at the top of this age range [14–17]. Evidence suggests that women over 45 are more likely to make the decision to stop attending than younger women [5, 8], to cite past traumatic experiences as a reason for non-attendance [4, 18, 19], and to experience the screening procedure as more painful [20]. Current evidence predicts a potential rise of more than 60% in rates of cervical cancer among older women by 2036 [21], suggesting an urgent need for targeted interventions to engage women in this cohort with home testing and cervical screening.

The impact of initiatives to encourage screening uptake is often low, localised or short term [7, 22–24]. In the European literature, interventions are largely task-focused, based on raising awareness by altering the content or source of information provision [2, 3]. Evidence from Africa and America suggests that consciousness-raising alone, while increasing women’s knowledge and awareness of the benefits of screening, does not necessarily translate into action [7, 24–26]. Engagement with screening requires behavioural change, and behavioural change is shaped by social and environmental context. Successful interventions beyond Europe have often developed around community education initiatives, and demonstrate how stakeholder involvement in intervention development can tailor interventions to fit local social and cultural contexts [27–29].

In the UK, Medical Research Council (MRC) guidelines for complex interventions [30] and National Institute for Health and Care Excellence guidelines [31, 32] emphasise the need to ground behaviour change within a theoretical framework. The explicit use of theory also allows us to understand the mechanisms of influence of such interventions and to replicate these [33]. Systematic review evidence demonstrates the effectiveness of the application of theory in this way [34–36]. Studies which have used behavioural theories to develop their interventions have shown more success in increasing screening rates [37, 38]. Crucially, these interventions take social determinants into account [3, 39] – those that influence women’s attitudes and health beliefs, including, for example, factors shaping women’s past experiences of screening and perceptions of risk. Many studies cite the use of theory to identify methods of behaviour change, but fail to describe in detail how theoretical constructs are transformed into intervention content [40–43]. Transparency about this process will broaden the toolbox for future intervention development, and enable more effective evaluation [33].

In this paper, our aim is to describe how barriers and facilitators to attending cervical screening, identified in qualitative data from a primary research study grounded in a constructionist epistemology [44], were categorised into theoretical constructs and used to identify appropriate behaviour change techniques. We then describe the stakeholder co-design of the content and mode of delivery of two pragmatic interventions: a service-user leaflet and a video animation for practitioners, for use in primary care (doctors’ surgeries and associated health networks) in the UK.

## Methods

### Study design and setting

The raw material for intervention development took the form of a data set from a qualitative study [44] conducted immediately prior to stakeholder co-design workshops. We selected the Theoretical Domains Framework [45] as the theoretical basis for our study as it synthesises all published models of behaviour and behaviour change, offering us a comprehensive means of understanding environmental, social, cultural, institutional and individual practice behaviour determinants. The framework uses language accessible to non-psychologists, giving it utility in the stakeholder co-design process, and once determinants are categorised to the framework it offers a pragmatic means of selecting the behaviour change techniques that are most likely to be effective [46]. The TDF has been tried and tested in other areas of health care [47–49] to inform interventions for both practitioner [50] and service-user [51] behaviour change.

Strategy for the analysis was formulated by the project steering team (all authors). BCT theory was applied by conducting secondary coding of the qualitative data set to draw out quotations describing barriers and facilitators of attendance; similar quotations were pooled to create a set of representative barrier and facilitator statements in a collaborative session involving three members of the research team (AB^1^, JD, HC). AB^1^, HC and JD are female researchers with PhD-level research methods training, each with applied health research experience spanning 10 years or more; JD is an implementation science specialist.

Barrier and facilitator statements were then categorised using the TDF to identify key domains [45], and the behavioural change techniques associated with these domains [46]. The barrier and facilitator data were presented to stakeholders by AB^1^, HC and JD in one lay focus group (FG1) and by AB^1^ and HC in two practitioner focus groups (FG2, FG3) convened in 2017 and 2018 in the two urban districts involved in the primary interview study. Focus groups were audio recorded, transcribed verbatim and anonymised; recordings were placed in secure data storage at the University of Hull. The focus groups formulated target behaviours for two interventions (one for service-users, one for practitioners), and designed intervention content based on the behavioural change techniques associated with key domains identified using the TDF. Interventions were then developed by the research team based on the focus group discussions, intended for implementation via primary care networks (general practitioner surgeries) in the UK.

### Sampling and recruitment of stakeholders for intervention development

FG1, which took place at the University of Hull, was convened by the research team from service-users interviewed as part of the qualitative study [44]. Participants from the previous study were asked at the end of their interviews whether they wished to take part in the co-design of an intervention; the majority declined and were not asked to give a reason for declining. Five service-user interviewees between the ages of 55 and 64 volunteered to assist (two had stopped attending for screening, two delayed attendance for complex reasons, and one attended regularly). The practitioner focus groups (FG2 and FG3) took place at primary care premises in two towns in the north of England serving areas with a high degree of deprivation. Both groups were recruited by three practitioners interviewed for the qualitative study, and included 11 further screening practitioners from their local primary care networks. FG2 involved four GPs and four practice nurses; FG3 included one GP and five practice nurses. All participants for focus groups were female.

### Intervention development procedure

**Fig. 1:**
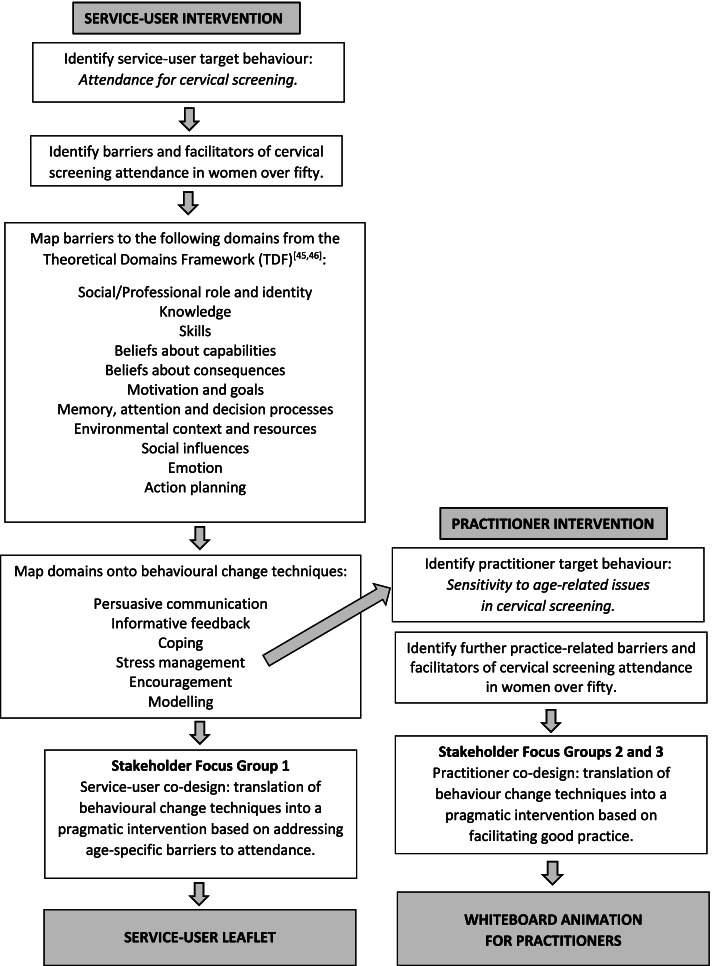
Intervention development flowchart

The target behaviour specified was attendance for cervical screening in women over 50. Intervention development subsequently involved three stages: the recoding of qualitative data to produce a set of barrier and facilitator statements, the categorisation of barrier and facilitator statements into domains following the TDF, and service-user and practitioner focus groups to facilitate the stakeholder co-design of intervention content from both perspectives. See Fig. 1 for a flow diagram of procedures.

#### Stage 1 – secondary coding of qualitative data set

The data set from the primary qualitative study focused on experiences of cervical screening in women over 50, and practitioner experiences of conducting cervical screening with women over 50. The thematic coding template developed in the original qualitative study was used as a guide to draw out statements representing barriers and facilitators of attendance (AB^1^). Themes exploring women’s difficult previous screening experiences, myths and misunderstandings surrounding screening, and the challenges faced by practitioners contributed data representing barriers. Themes exploring family health talk, sexual health and relationships, and history-taking and rapport-building during appointments contributed data representing facilitators. Less prevalent barriers and facilitators were noted where they appeared elsewhere in the data – for example, knowledge deficits and environmental influences (such as perceived difficulties with screening equipment, where women associated the procedure with a metal speculum and scraper used in earlier decades rather than the present-day plastic speculum and brush).

Multiple quotations from the qualitative data represented similar concepts. The statements were read by three research team members (AB^1^, JD, HC), and in a full day collaborative analysis session, the team pooled similar quotations into two sets of summary statements representing barriers and facilitators in preparation for stage 2 (see Table 1 for examples).

**Table 1:** Examples of data contributing to summary statements, and of TDF domains matching the statements

Examples of data	Examples of summary statements: key barriers	TDF domains
*‘I just wonder if it’s perhaps in a family history of when, like, I suppose if there’s been one or more people, like two or three people in your family that have had it, I would imagine that that would actually raise your risk of it... Maybe people that have been sort of a bit promiscuous, prone to infection, something like that might trigger it.’* Attender (LS24^a^) *‘I’ve been with my husband since I was 18, we’re still together. I’m pretty certain he’s monogamous... I’m certainly monogamous, so I don’t feel like I’m at risk.’* Attender (LS17) *‘She [practitioner] should have sat me down in the first place, ascertained any problems around the smear – what do I understand about it? She never did any of that, it was just a question of the mechanics of it. So I, I want an explanation.’* Non-attender (LS2) *‘I think they just feel that if it was going to happen it should have all have happened by now – and that’s it for me now, just, my ovaries are switched off, it’s, everything’s winding down or wound down and that’s it.’* GP (HCP9^b^)	My risk of getting cervical cancer is low. I don’t know why I still need a screening test.	Knowledge.
*‘I might be just in my sixties now but I mean I’m still… I’m quite a young 60, erm and I’m still having a sex life... I’ve been pushed on the scrap heap, they don’t wanna know!’* Attender (LS19) *‘I’ve been wondering at the, the diff, the different changes now, in patient’s, in people’s lives because there’s a lot of ladies and partners splitting up in their forties and fifties... And then there’s a lot more new partners... Maybe, do they see it that actually they don’t need, is it because they don’t need sexual protection because they’ve gone past the menopause? ... I’m beginning to, wondering if that is it, is that, if that’s the reason why it’s changed, because of the dynamics that have changed and people getting older, they’re no longer staying to that one partner.’* Practice Nurse (HCP5)	Doctors and nurses think no-one has a sex life after 60.	Role/Identity.
*‘I can just feel it now, I can just, you know, remember it in my mind, it’s just like putting something really dry, oh, up something that’s all [laughs] sunk in, and it just doesn’t work, you just can’t do it.’* Non-attender (LS16) *‘It wouldn’t surprise me if, if a lot of the over fifties don’t attend because they’re not having regular sex, and therefore they perceive that it would be difficult, or sex is difficult.’* GP (HCP14) *‘I get uncomfortable because my body, my hip locks on me... Well usually you have to lie on the bed don’t you and hunch your legs right up and open? I can’t expand my legs... they pulled me right down to the edge, had like one of the nurses there and I had to put my feet on her as far up, and I mean it, it was painful.’*Attender (LS21) *‘The laying down, that’s not the problem. It, it’s the actual physicalness of putting your ankles together. And, and, and opening, opening your knees. It’s your joints.’* Non-attender (LS15)	Inserting the speculum is painful because everything feels too dry. I can’t get in the right position for the test any more, because it causes physical discomfort.	Beliefs about capabilities.
*‘When you get to a certain age – age is a factor, illness is a factor – but age is a factor that you become more, more of a sponge to what’s going on in the world, and there’s not much you can do about dying or preventing your own death, so it becomes less important.’* Non-attender with multiple sclerosis (LS4) *‘I got to 50, I went and had my mammogram and they found a lump, so I had to, so it just put me off going to having anything done, I just don’t want to know, if I’ve got anything wrong I don’t want to know.’* Non-attender (LS23) *‘I don’t think you can do anything. I think if you’ve got something, you get it.’* Attender (LS3) *When I became ill, to be honest that was the furthest thing from my mind... it’s still too much, it would be too much for me stresswise to cope with if. If I came and had a smear and got a negative, erm, feedback.’* Non-attender with arthritis and circulatory problems (LS15)	I have too many other health issues – if the test picked up abnormalities, I wouldn’t want to go through treatment anyway. There’s nothing I can do to stop myself getting cervical cancer. If something is wrong, I’d rather not know, I wouldn’t cope.	Beliefs about consequences.
*‘I had gone when I started with the problems after my menopause, to see a lady doctor at the surgery, and to be honest I felt, I felt that she thought I was just being, not stupid, but it wasn’t important the fact that I had no sexual intercourse or anything like that and the marriage was breaking down. And she, “Oh, if that’s all that’s bothering you!”, sort of thing. And she was an older lady doctor... I just felt after she’d said that, God I shouldn’t be troubling the doctors with things like this.’* Non-attender (LS5) *‘I think it’s quite bad really... it’s 65 then you’re kind of cut off... not everyone’s sort of past their sell by date and finished with are they really?’* Attender (LS21) *‘If I speak to women who have menopausal problems or pain with sex, which often you see people, and anyone who’s menopausal to be honest, I, if, if they’re coming to talk to me about the menopause, I will raise that and say actually use the oestrogen cream and lots of moisturiser. That’s what we should be telling everybody... we should be encouraging any women, over 50 to, to treat that as essential part of their healthy life.’* GP (HCP14)	I’ve had problems with dryness since hitting the menopause, but my GP told me these things aren’t worth addressing at my age.	Motivation and goals.
*‘I’d have to have a reminder that, you know, you haven’t been for this examination for a while... I’ve just put it to one side and forgotten I’ve got it... I tend to, I don’t mean conveniently forget because I don’t, I just forget, you know... months later I’m going through the bottom of my bag [of paperwork] and thinking – ooh, what’s this?’* Attender (LS8) *‘They’ve put it in their pile of letters and the day’s gone on and they’ve forgotten or they’ve rung up and they couldn’t get through to the GP surgery and it, it gets forgotten. And then something happens and nobody follows it up and that does happen in, in some practices. And if that happens it can go on and on for years. And it’s, and it’s modern, busy life, it’s understandable.’* Practice Nurse (HCP17) *‘Time fades, doesn’t it really? And I think... if they were to come back after 5 years when they should have come back, whatever it were that triggered it in the first place is soon forgotten, unless there’s some other trigger factor that happens in the meantime.’* Practice Nurse (HCP21)	I put screening invite letters in my ‘to-do’ pile and they just get forgotten.	Memory, attention and decision processes.
*‘We a good rapport with each other… when she actually said “Oh, have you had your smear test letter?” I said “yeah”, she said “Well let’s book you in”. I’d gone for erm a blood pressure test... So each time I got one, I said “Oh I’ve got my letter” when I’d go for a blood pressure test, she’d book me in rather than me waiting for the receptionist to buff you off and everything else that they do.’* Attender (LS13) *From the start [laughs] it just seems... little sort of avenues off. Never mind getting the appointment, never mind actually on the bed and doing what you need to do... The stress I think of having to check in at reception – no-one’s there, then she’s logging in, I’m thinking “For goodness’ sake, woman!”... And then, to top it all, [laughs] I know it’s a Well Woman Clinic, and she goes, “Oh, it’s important to be, erm, mentally alert!” – “Yeah, I do work in a [customer service] environment, I’m mentally alert, yeah”... I feel oh, just keep, I feel it drags me down. I know I shouldn’t say, but I feel the whole procedure of reception, seeing different people, different nurses.’* Non-attender (LS25) *‘Well I suppose if you’ve got a 20 min appointment, somebody’s not turned up, yeah, you could ring them. But then equally then that can make people feel really bad if they’ve forgotten. [laughs] And we’re not out, I’m not out as a blame culture.’* Practice nurse (HCP20)	Communication with my GP practice is important, and it’s not always easy.	Environmental context and resources.
*‘I’ve had smears from doctors who treat you like a slab of meat... that turns you off a little bit.’* Attender (LS20)*‘It can be a very intense sort of space… women just wanna get it over with… it’s a space that can be quite emotionally charged… it’s so emotional, this smear test, and I think that’s got to be tackled.*’ Non-attender (LS2)*‘I felt as if she was ramming something into me and it was just extremely, you know, personal and uncomfortable. And I I felt afterwards I’m not going to her again.’* Non-attender (LS1)*‘The first horrid one I had... she had her back to me for a while, she’d left the thing [speculum] in... I said, “I’m shaking, I can’t stop my legs shaking, it hurts like mad!”... it was as if she didn’t hear me and she’s carrying on, and to me it was like some torture chamber or other.’* Non-attender (LS16)*‘Ladies of a certain age might think to themselves it was an abusive experience, so therefore that could be a reason why some women are reluctant to go these days... I was terrified. I didn’t like my GP, he was – won’t mention any names for confidentiality purposes – but erm, don’t want to put this in too strong a terms but he made me very uncomfortable.’* Attender (LS17)	Whenever I’ve had intimate examinations in the past, I’ve felt uncomfortable/ severely distressed.I find the screening procedure intimidating and/or impersonal.Screening reminds me of past traumatic experiences.	Emotion.
*‘Big red letters: “No smear! No smear! No smear! Offer smear! Offer smear!” No one ever discussed why I wasn’t going to have it with me. I thought, I’m not bringing it up. [laughs] I don’t bloody want it in the first place but, yeah. It was never discussed. Never discussed.’* Non-attender (LS2) *‘No-one’s ever asked me at the surgery where I was before about why I didn’t want to do anything or – not that I resent anything – but why, well basically any options... they just took it as mainstream, yeah, you’re going to come for a smear.’* Non-attender (LS25) *‘Ask the question. So remind them first of all that they need it, and then ask them the ‘Why’ [they don’t attend] in a way… and be prepared to do something about it.’* GP (HCP1) *‘They can treat it... they can take it away by scraping or, you know, whatever, so that that really is my knowledge of it... so yeah, daughters... they’re more aware of things like that... When you’re growing up in the seventies, you weren’t taught anything like that so it’s up to you to go out there and find out... but again not always, erm, people there to talk to is there? ... So but yeah, daughters, that’s why I know a little bit more about it... because they both had abnormal cells as well.’* Attender (LS18) *‘Occasionally you will get a couple that are kind of over their fifties. More often than not... their daughters have pushed them into it, because the daughters are kind of coming up to that age for it and they’ve been for theirs, and if they know their mum’s out of date... I have had a couple saying, “Oh my daughter came for hers last week and told me I had to book in for it”.’* Practice Nurse (HCP19) *‘One of my friends... she didn’t go for a smear test for years... she’d had letter after letter, and then she said “I am absolutely terrified”, and I said, “Well I’ll come with you” and we was in the, in the hospital waiting and she put her coat on and started walking. “Where you going?” She said “I can’t stay”. I said “Yes you can, you can, it’s your body and you need to know that you’re clear, do you want to end up bad with cancer or, or something and end up dying with it?” And she went “No”. And I said “Well, that’s your answer”. She was fine, and she still goes now.* Attender (LS21)	No-one at my GP surgery ever has ever bothered to ask me why I don’t go for screening. My daughter persuaded me to go for screening. Friends my own age persuaded me to go for screening/I persuaded a friend to go.	Social influences.

^a^*LS* Lay stakeholder, ^b^*HCP* Health care practitioner

#### Stage 2 – categorisation of barriers and facilitators into theoretical domains

For this project we chose to use the consensus matrix proposed by Michie et al. [46] for its clarity and utility. This provided a clear protocol for linking TDF domains with behavioural change techniques. This work has been developed further by Michie et al. [52] and Carey et al. [53], and intervention developers can now take advantage of an online Theory & Techniques Tool [54]. Summary statements representing barriers and facilitators were categorised under the following constructs from the TDF: knowledge, skills, role and identity, beliefs about capabilities, beliefs about consequences, motivation and goals, memory/attention/decision processes, environmental context and resources, social influences, emotions and action planning. Matching data with domains was a subjective process involving discussion and negotiation among the team until consensus was reached.

#### Stage 3: stakeholder focus groups

Focus group 1 involved service-users, focus groups 2 and 3 involved practitioners; each focus group lasted 1.5 hours.

##### Service-user focus group

In focus group 1, patient stakeholders were introduced to the concept of identifying the target behaviour (cervical screening attendance in women over 50). The research team presented barriers and facilitators data and explained the process of linking these with the domains of the TDF. Behavioural change techniques for addressing the key identified TDF domains were then introduced by the team’s behaviour change specialist (JD) (see Table 2). Photographs from popular advertising focusing on lifestyle and health were used to assist an explanation of the principles of behaviour change, and to provoke thought about the focus of an intervention (for example, images of people over 50 engaging in ‘healthy’ activities, and of interactions between health care professionals and patients). Stakeholders were encouraged to discuss their ideas for intervention content based on the relationship between the target demographic to which they belonged (women over 50) and the qualitative data statements. Potential modes of delivery were brainstormed with APEASE criteria in mind: affordability, practicability, effectiveness, acceptability, safety and equity [55].

**Table 2:** Developing the content of the patient intervention using theoretical constructs from Michie et al. [46]

Behavioural change technique associated with key TDF domains	Application of theory to intervention content
Persuasive communication.	Warm and empathetic tone.
Information regarding behaviour/outcome.	Question and answer format, correcting myths and misunderstandings about screening/its outcomes: • distinguish myths from facts; • address age-related questions about the screening process.
Stress management.	Illustrate importance of rapport with practitioner/sensitivity of practitioner to experiences of women over 50.
Modelling/demonstration of behaviour by others. Social processes of encouragement, pressure, support.	Use social influences meaningful to women over 50/role modelling of discussing and attending screening by people they can relate to.

##### Practitioner focus groups

In focus groups 2 and 3, the same barriers and facilitators of attendance were presented in categories, shaped by the service-user focus group discussion of practitioner challenges (‘patient’ barriers, practice barriers, and facilitators of good practice). Stakeholders were asked to identify key challenges in the practice of cervical screening with women over 50 in relation to the barriers to attendance, and to match facilitators to the challenges in a way that characterised ‘good practice’, evidencing sensitivity to age-related issues connected with cervical screening. Key elements of these discussions are summarised in Table 3.

**Table 3:** Examples of barriers and facilitators from the data which fed in to good practice recommendations

Barriers informing outcome		Outcome
Patient barriers	Practitioner barriers	Good practice: key challenges
Examples from data: *• Non-attenders’ perception of poor/impersonal communication from practitioners.* *• Attender and non-attender experiences of problems discussing sex and relationship changes associated with aging with practitioners.* *• Experiences of screening tests from previous decades becoming a ‘guiding light’ (non-attender interviewee) for decisions about attendance in the present.* *• Lack of practitioner sensitivity to pain and discomfort caused by vaginal dryness.* *• Difficulties keeping appointments which have to be booked far in advance.*	Examples from data: *• Lack of networking between practice nurses who carry out cervical screening.* *• Difficulties in making older women comfortable when they have menopausal or mobility issues; lack of continuity with patients in addressing difficulties.* *• Difficulties with equipment (table height not adjustable, lighting inadequate, etc).* *• Diversity and strength of expectations among older patients – may need pragmatic or ‘businesslike’ (attender interviewee) approach, or empathetic and understanding approach, dependent on screening history.*	1. How to identify and communicate with non-attenders. e.g. *Draw on person-centred communication procedures (non-judgemental language/open approach); facilitate networking between practice nurses around non-attendance.* 2. How to make appointment protocols flexible in a way which encourages attendance among older women (advice which can be customised by each GP practice dependent upon capacity). e.g. *Offering a pre-screening appointment to discuss issues; matching patient with appropriate nurse based on key issues.* 3. How to develop rapport with older women attending for screening. e.g. *Examples of ‘history-taking’ techniques – how to talk to older women about sexual or relationship difficulties connected with screening avoidance; recognising importance of previous screening experiences; asking women what they know about their anatomy (*i.e. *previous experiences of gynaecological exams evidencing difficult positioning of cervix).* 4. How to tailor the screening process to older women’s needs. e.g. *Provide instructions for addressing gynaecological issues such as menopausal dryness, mobility issues/problems associated with chronic illnesses. Instructions about positioning women in different ways for the procedure, and use of speculums/lubrication.*

Transcripts of the focus groups were summarised to guide the written intervention content, which was structured to fit the mode of delivery recommended by stakeholders. The translation of qualitative data into intervention content is described in detail below.

## Results

The majority of the barrier/facilitator data clustered beneath three TDF concepts: *beliefs about consequences*, *social influences* and *emotion*, and smaller clusters of data corresponded with *beliefs about capabilities* and deficits in *knowledge*. Examples of data mapped on to the domains are given in Table 3. The mapping framework from Appendix B of Michie et al. [46] was used to match the three most prevalent TDF concepts with appropriate behaviour change techniques: *persuasive communication* and the provision of *information regarding behaviour/outcome* to address beliefs about consequences, *stress management* to address difficult emotions, and *role modelling* and *encouragement* to harness social influences (see Table 2).

### Service-user stakeholder group

Stakeholders were introduced to behaviour change techniques related to the processes described above, and how these might be harnessed in the development of intervention content (Table 2). The target behaviour was attendance for cervical screening.

#### Development of intervention content

There was a strong consensus that the provision of information for women over 50 should focus on questions about screening protocols or uncertainties about continuing screening, and that as ‘patients’, women do not always know how screening might change with age, or what questions they can legitimately ask:

*...if you were going to do, for example a leaflet, sorry, I'm sort of thinking outside the box really... about practitioners or the nurses with the speech bubble, you could sort of do a patient asking ‘Does it hurt?’ ... ‘Will I bleed?’ ... if they can open up the leaflet, that won't be on the front page obviously but that'd be inside so you might reassure people... I didn't know that there was even a brush that went in me... I didn't even know that, I just thought it was like a little ramrod went in you really, I didn't, [laughs] I don't even know.* Stakeholder 1, FG1

Stakeholders stated that the questions included needed to be uniquely pertinent to the experience of aging and menopause. On reconsidering suggested modes of delivery after this discussion, a printed leaflet asking and answering age-related questions about screening was suggested as the most practical way of addressing these concerns, with content guided by experiences of intimate examinations and misunderstandings about screening among women over 50 drawn from the barriers and facilitators data.

**Fig. 2:**
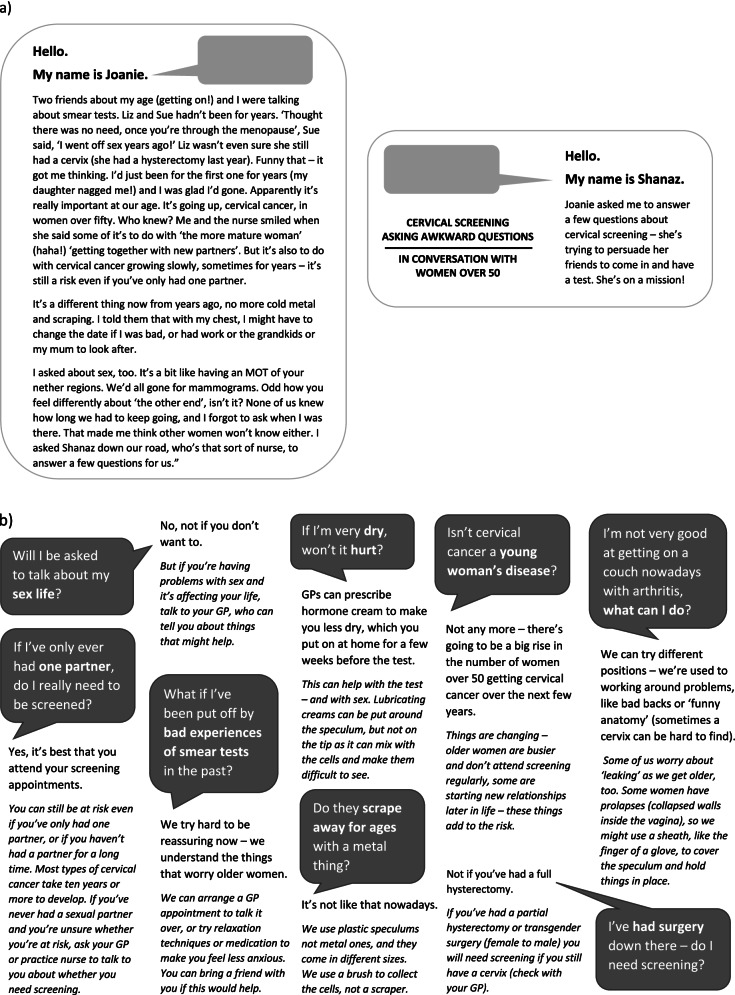
**a** Introducing a screening story and service-user/practice nurse interaction on the service-user leaflet. **b** Examples of question-and-answer text on the service-user leaflet

In considering how the visual elements of the question-and-answer section would work, stakeholders emphasised that rapport between women and screening practitioners was central among the facilitator statements. Among the visual material provided to provoke discussion, stakeholders chose a photograph of a nurse and patient to represent the importance of personal communication and the building of rapport: *‘there’s like some sort of relationship, their heads are right close together’* (Stakeholder 2). The consensus was reached that questions and answers could be presented as a conversation between a practice nurse and a ‘patient’, and that this should be introduced by a service-user story created from the interview data in which a woman over 50 is described talking with friends about cervical screening, to role model attendance behaviour. See Fig. 2 for the service-user story and examples of question-and-answer text.

Stakeholders perceived stress management as part of the practitioner’s role, citing barriers to attendance which described difficulties in communication with service providers, and emphasised the need for confidence and reassurance: *‘I don’t do doctors any more, just forget it, you know, it causes aggravation... I’ll just stay at home, I’ll just Google, it’ll be fine!’* (Stakeholder 1). Discussion of strategies for stress management led to the identification of the target behaviour for a practitioner intervention: the demonstration of increased sensitivity to age-related issues during the screening process (which included appointment making and pre-screening conversations as well as the test itself), as a way of managing the stress that can be experienced by women over 50 in relation to cervical screening.

#### Mode of delivery

Service-user stakeholders considered the range of contexts in which information about cervical screening in women over 50 could be effectively disseminated. Ideas included printed messages on supermarket till receipts, leaflets, open days at doctor’s surgeries, and the use of role models via media campaigns. Focusing on the APEASE criteria [55], in particular on practicability, it was felt that women’s need for privacy could be reflected in a concertina-style leaflet, folded up to hide the content, to fit inside a purse or pocket. Distribution was to occur via primary care or via suitable community venues.

### Practitioner stakeholder groups

In preparation for the practitioner focus groups, barrier statements were categorised under *Challenges to attendance* and divided into the subcategories *‘Patient’ barriers* and *Practice barriers.* To guide the discussions, data statements were summarised into four key challenges related to reducing the stress that can be associated with cervical screening for women over 50 (see Table 3): two challenges emerged at the organisational level (1 and 2) and two at the individual practitioner level (3 and 4). Facilitator statements offered examples of potential good practice in each area.

#### Development of intervention content

The four challenges were discussed in relation to the local demographic contexts of individual GP practices, and developed in more detail to inform the intervention content. Appropriate communication (challenge 1) was linked by practitioners with proactive contact with non-attenders, introducing cervical screening opportunistically during other health consultations, and allowing responsibility for the decision to rest with the patient. Flexibility (challenge 2) included allowing for pre-screening appointments to explore difficulties, and maintaining individual nurse-patient relationships across multiple screening appointments where possible. The development of rapport (challenge 3) was connected with taking time to explore women’s past experiences:

*That, that is the key and the crux to being able to get a successful smear and for that lady to come back and have that confidence in you, is, is the history taking, I think that’s the most important thing.* (Stakeholder 1, FG3, Practice Nurse)

*It’s listening to your lady, ask, actually ask them why, why haven’t they come? What’s the problem? What can we do to help? It’s just listening and getting a rapport.* (Stakeholder 3, FG3, GP)

Suggestions for tailoring the screening process to women over 50 (challenge 4) included increasing practitioners’ knowledge of alternative positioning to accommodate mobility issues, and offering preparative appointments prior to screening to allow the prescription of oestrogen cream to resolve dryness or medication to counteract anxiety, if appropriate.

#### Mode of delivery

An initial proposal of a laminated A4 sheet detailing the good practice points was rejected by practitioners as unsustainable as it was likely to be overlooked or become lost. Training for cervical screening was seen as onerous by both practitioner groups, and they requested an intervention that was focused and short. The consensus was that the best form of delivery would be a short audio-visual that could be watched on a mobile phone in work breaks, or on a tablet or computer, that could also be embedded in the current mandatory on-line training course for cervical screening practitioners in the UK and rewarded by credit contributing to continuing professional development (CPD).

## Production of the interventions

### Service-user intervention

#### Content development

The leaflet content comprised of a series of ‘patient’ questions and practitioner answers based on issues arising from the interview data to address the challenges in cervical screening for women over 50, and to overcome myths and misunderstandings about the screening process in evidence among the target population. Figure 2 shows examples of questions developed during the patient stakeholder focus group. Answers to the questions were drawn from facilitator data and examples of good practice discussed in practitioner focus groups.

#### Mode of delivery

A 300 mm × 235 mm leaflet was produced, targeted at women over 50. The leaflet folded up into a credit card size between two card covers (84 × 54 mm).

### Practitioner intervention

#### Content development

An 11-minute audio script was developed by AB^1^ in consultation with the research team. Table 4 illustrates key issues arising in the focus group discussions that were included in the script. Based on discussions in the stakeholder focus groups, a decision was made to focus the animation around a conversation between two female friends over 50 (one a screening attender, the other a non-attender), using quotations from the interview data to construct a dialogue which systematically illustrated barriers to and facilitators of attendance. The storyline moved through the women’s lifecourse, from their twenties to their sixties, to mirror the ‘history-taking’ described by Stakeholder 1 in FG3, above. The narrative explored the experiences and challenges specific to cervical screening and the facilitators of good practice, as discussed in FG2 and FG3. A women’s health expert known nationally to practice nurses and GPs in the UK narrated an introduction to the conversation, and drew out key points for a call to action at the end of the animation. (See Additional file 1: Animation Script).

**Table 4:** How key issues from stakeholder focus groups converted into action points in the animation script

Good practice points	Areas of focus group discussion	Focus of animation script
1. Identify and communicate with non-attenders who are over 50.	• Link cervical screening with chronic illness reviews, carer reviews, etc. • Ring non-attenders directly about screening: listen, inform, explain. • Have regular practice meetings raising patients’ individual issues. • Raise awareness, address myths and misunderstandings.	**Introduction:** Professional expert on women’s health (General Practice) describes why and how the intervention has been put together. **Central section:** A conversation between two women over 50, voiced by actors, illustrates the challenges that cervical screening practitioners may face with this cohort. The dialogue follows a timeline of screening-related experiences from women’s twenties into their sixties, through the decades. Phrases drawn from the qualitative interview data are woven into the dialogue to illustrate the barriers and facilitators of attendance. The narrative explores: • misunderstandings surrounding the screening test; • different attitudes towards risk; • how experiences of intimate examinations in previous decades can affect attitudes towards screening; • how sex/relationship issues affect attitudes to screening; • how problems related to menopause and chronic illness can affect practical aspects of the screening test. **Close:** The women’s health expert summarises the key issues and states a three-point call to action: • **Prepare**: Address physical and psychological issues, build a network of professional support to develop your expertise. • **Listen**: Take patient history, build rapport, address psychological and physical challenges. • **Adapt**: Where possible and practical, take a flexible approach to appointment booking, and to screening procedures (e.g. positioning).
2. Make appointments flexible in a way which encourages attendance in older women	• Offer repeat appointments over time rather than one-off appointment. • Offer extended hours (dependent on capacity). • Offer screening opportunistically. • Network with other screen-takers in your GP practice. • Allow your patients to choose their screening practitioner.	
3. Develop rapport with older women attending for screening.	• Inform patients about how screening procedures have changed. • Proactively ask women why they do not attend. • Talk through the procedure, inform women in personal manner. • Encourage collaboration between older and younger practice nurses to talk through age-related issues. • GPs to be made aware of reasons for appointments in advance.	
4. Tailor the screening process to take older women’s needs into account.	• Discuss and address sexual difficulties caused by menopause and/or chronic illness. • Have all tools ready in advance, do not leave the room, actively problem solve environmental issues (e.g. broken door locks) in a timely manner. • Make plastic speculums standard. • Learn to ‘size’ women for appropriate speculum as they enter the room. • Allow women to insert speculum themselves. • Practice different positioning for older women to take account of mobility problems. • Have senior screening staff in attendance to offer practical advice. • Invest in rapport-building with colposcopy units to draw on expertise where screening is difficult.	

#### Mode of delivery

An 11-minute educational whiteboard animation for download on a mobile phone and dissemination on remote training platforms.

We are now looking to embed these interventions in the UK primary care setting via general practitioner surgeries and (for the practitioner intervention) online training for GPs and practice nurses as a supplement to training currently in place for cervical screening.

## Discussion

There is evidence that the use of behavioural change theory can increase the success of interventions [56, 57]. This approach has been used to develop a limited number of cancer screening programmes to increase the chances that knowledge will translate into action [3]. In this study, our intentions in using a theoretical approach were twofold: (1) to explore the determinants that mediate between thinking about attending for cervical screening beyond the age of 50, and acting on those thoughts, and (2) to use our findings to shape focused intervention content through stakeholder engagement. This discussion will explore the potential benefits and drawbacks of these processes.

The analytic framework of our primary study provided a guide to recoding our data into barrier and facilitator statements. Our interview study demonstrated that the determinants of screening attendance are not only shaped by the psychological and physical changes women experience as they age, but by relational aspects of the screening encounter – specifically, women’s interactions with GP practice staff, individual screening practitioners, peers and sexual partners. Themes describing emotional difficulties and misunderstandings about cervical cancer guided us towards barrier statements related to the existing cervical screening literature, themes describing practitioner challenges in the screening encounter provided additional barrier statements, and themes exploring women’s sexual histories and mother/daughter and patient/practitioner relationship-building provided the majority of facilitator statements.

In the original qualitative study, participants were not asked to interpret their experience through the lens of theoretical domains during the interview. Cervical screening was a sensitive subject, and interviews focused on eliciting interviewees’ experiences of intimate screening, to avoid leading the agenda surrounding attendance. We would argue that structuring interview schedules around the domains of the TDF [58] runs the risk of placing the agenda too firmly with the theoretical framework at the expense of exploring the main characteristics of the experience under question.

For our study, the free coding from the original qualitative study analysis aggregated data on barriers and facilitators as they emerged from stakeholders’ descriptions of experience. Given that barrier and facilitator statements are quantified when they are assigned to the TDF, the selection of salient domains to pursue with behaviour change techniques was driven by the elements of screening that interviewees chose to talk about in relation to our research question (‘How does aging affect women’s experiences of decision-making about attendance for cervical screening?’). This hybrid approach [59], with deductive theoretical coding informed by an initial inductive analysis, allowed the stakeholder perspective to remain central and drive the distribution of barrier and facilitator statements in a way which remained true to participants’ experiences.

Matching barrier and facilitator statements to the theoretical domains of the TDF was a subjective process involving collaboration and negotiation between the research team in face-to-face meetings. Where the placement of statements was contested, the team were able to reach agreement over which statements best represented which domains. Intervention development via focus groups allowed the team to present and discuss the results of this process with stakeholders. This provided a structure for stakeholder consultation, and an opportunity for ‘member checking’, with participants able to review and confirm which aspects of the team’s decision-making made sense to them [60–62]. It also enabled the research team to explore how intervention content and mode of delivery might resonate with its intended audience.

The original study on which this paper is based was conducted in 2016-18. The theoretical principles used in the study have developed considerably – not only have citations of the TDF increased exponentially since the framework was first created, but the pace of change and refinement has been fierce, leaving published study methodologies lagging behind theoretical developments [46, 51, 53, 55, 63]. Further exploration of behavioural constructs have been systematic and methodical, and the protocol for developing intervention content from qualitative data described in this paper is replicable using the more recent Theory and Techniques Tool [54] to map the TDF domains on to behavioural change techniques.

### Strengths and limitations of the study

Recruitment for the original qualitative study lacked diversity in terms of the ethnicity. Study material was distributed to all women on GP lists who were more than 1 year overdue for cervical screening, but all volunteers were white British. The original study did not record the ethnicity of those who were approached for participation, only of those who volunteered for interview (potential interviewees were recruited by practitioners and their details passed on to the research team, with their permission, to maintain confidentiality). While the practitioner focus groups for intervention development were more ethnically diverse, patient data considering demographic and ethnic diversity, while present, was sparse. This limited the exploration of the intersection between ethnicity and age.

Demographic homogeneity is often encountered in stakeholder consultation with older people [64], and our efforts at inclusivity were inevitably guided by the voluntary response to the interview study. We believe that the *methodology* of intervention development used in this study was recriprocal and iterative, and would work with other similarly homogeneous groups in different contexts. In locations where the community-based participatory approaches described in our introduction are not viable for reasons of time and cost, smaller studies with culturally homogeneous groups using behavioural change theory could highlight aspects of commonality and divergence and elucidate aspects of demographic diversity in this cohort of women over 50.

The key strength of the study was the inclusion of the practitioner perspective. The practitioner/service-user relationship is a crucial aspect of the health service context, and this interrelationship of perspectives was a key focus of the qualitative data, which reflected the central importance of history-taking, relationship building and rapport necessary for women’s comfort with the cervical screening process. The centrality of such relationships is also evident in community-based research – for example, in the engagement of community health navigators to facilitate screening [65]. The practitioner focus groups in our study raised cultural issues surrounding the intimacy and potential invasiveness of the cervical screening test, and discussions explored how culturally specific research using similar methodologies might further inform practice in demographically diverse areas.

## Conclusion

Despite the broadening literature describing the use of behavioural theory to develop interventions, there is ongoing debate about the efficacy of this approach [43]. In the area of cervical screening, existing interventions to encourage attendance are not easily comparable – reviews evidence a great deal of heterogeneity in study designs and a lack of description of the foundations of intervention content, and often fail to include lessons learned from the successful engagement of stakeholders in community based approaches. We would argue that the use of theory can focus the intervention development process and keep intervention content aligned with the priorities of stakeholders. The Theoretical Domains Framework, in combination with the Theory and Techniques Tool [54], offers a stepwise, auditable protocol for developing intervention content which is amenable to clear reporting and replication in different local contexts. The detailed reporting of protocols for translating qualitative research into intervention content is imperative to achieving transparency, consistency and quality in the material that we chose to test and evaluate. It will also allow a deeper exploration of how stakeholder perspectives might successfully contextualise interventions for specific local populations.

## Supplementary Information

**Additional file 1.** Practitioner intervention: Animation script.

## Data Availability

Examples of data generated during this study are available within the article and Additional file 1; the original qualitative dataset on which the study is based, and the whiteboard animation, are available from the corresponding author on reasonable request.

## Abbreviations

APEASE: Affordability, practicability, effectiveness, acceptability, safety and equity
BCT: Behaviour change theory
CPD: Continuing professional development
FG: Focus group
GP: General Practitioner
HCP: Health care practitioner
HPV: Human papillomavirus
LS: Lay stakeholder
MRC: Medical Research Council
TDF: Theoretical Domains Framework
