# Supplementary material for: Translating qualitative data into intervention content using the Theoretical Domains Framework and stakeholder co-design: a worked example from a study of cervical screening attendance in older women
Source: BMC Health Serv Res. 2022 May 6;22:610. doi: 10.1186/s12913-022-07926-2 (PMC9074234; doi:10.1186/s12913-022-07926-2)
Supplement: Supplementary file 1 — Additional file 1. Practitioner intervention: Animation script. [file 12913_2022_7926_MOESM1_ESM.docx]

| **Animation script: Practitioner intervention** | |
| --- | --- |
| **NARRATOR (WOMEN’S HEALTH EXPERT): INTRODUCTION**  Hello, my name is [women’s health expert]. I am a GP [general practitioner/physician], and I have a special interest in women’s health.  In the years ahead, we’re facing a rise of more than sixty per cent in the number of women over fifty getting cervical cancer, and by 2036, the highest incidence will be among women aged 50 to 59. Women who are currently in this age group are not protected by the HPV vaccine, so it’s vitally important that we do everything that we can to support them in attending their cervical screening appointments.  A research project at Hull York Medical School, funded by Yorkshire Cancer Research, has shown that some of the reasons that women over fifty find cervical screening difficult are very different to the reasons given by younger women. In this short video, we’re going to listen to Eleanor and Joanie, who are going to talk together and share their very different experiences of cervical screening through their lifetime, from their twenties to their sixties. When we’ve heard their stories, we’ll then look at how some of Eleanor and Joanie’s concerns about screening can be addressed by us, as health care practitioners. | |
| **PATIENT ROLE-MODELLING CONVERSATION** | **GOOD PRACTICE POINTS 1-4** |
| **LIFECOURSE DIALOGUE BETWEEN ELEANOR (SCREENING ATTENDER) AND  JOANIE (NON-ATTENDER)**  *[IN THEIR TWENTIES: Early experiences of screening]*  **Eleanor:** I remember smear tests when I was in my twenties...those plastic things they put inside you these days are much better than those huge old clanking metal ones they used to use...My sister used to call it ‘the clamp’. I remember they used to keep those things in a bucket afterwards. You felt a bit cattle-like, really, in the old days.  **Joanie:** So they don’t use those metal things any more?  **Eleanor:** No, no. You can get them in different sizes now, too.  **Joanie:** Oh really? In those days, if you just went to the doctor’s surgery knowing that you’re going for a smear, nobody talked to you about what was gonna happen, they just did it. When I was in my thirties I had a really bad smear test. It was a lady doctor, too. I felt like she was ramming something into me...it was like being assaulted. All the people in the waiting room will have heard me shouting. That’s what put me off going – I just stopped going for the test.  *[MOVING INTO THEIR THIRTIES: Different responses to intimate examinations during pregnancy]*  **Eleanor:** I found it a bit easier after I’d had my kids – I think once you’ve had children you lose all your inhibitions, don’t you? It’s less of a worry isn’t it, people furtling about down there...you’ve gone through all that shy bit.  **Joanie:** Yeah. They did as they liked in those days though – when I first found out I was pregnant, the smear was just part of the booking in process. I told them I’d just had one done, ‘Well it’s part of the booking, we’re doing it anyway!’ It was quite abusive really...anybody other than your sexual partner having any contact with your genitals, it’s quite alien isn’t it? Really, it’s not like shaking hands with people, let’s be honest! [laughter]  *[IN THEIR FORTIES: Feelings about the body changing with age]*  **Joanie:** ...and when I was young, I had smears from doctors who treated me like a slab of meat, really unpleasant. You get a piece of blue paper, “Oh, and cover yourself up with that!” – I mean, a piece of blue roll, well it doesn’t cut it! [laughter]  **Eleanor:** As you get older and fatter, you don’t really want to see your own bits and bobs, do you? When you’re laid there sometimes you could just sort of like, die, especially if you’re overweight... last time it was OK – the nurse who did it was about my age...she wasn’t judging me , if I was thin, fat or whatever, she was a bigger lady herself...she put me at ease and I didn’t feel embarrassed or anything. I’ll ask for her again next time I think.  *[MOVING INTO THEIR FIFTIES: Life becomes full of responsibilities towards other people]*  **Eleanor:** Have to admit, I didn’t go for about ten years when I was in my forties and fifties. I had my parents to look after...started to think at that point, I cannot fit anything else in, I just can’t, it’ll have to wait...poorly parents, children, grandchildren, there’s so many other things to look after you don’t tend to look after yourself as much as you should do, do you?  **Joanie:** No. I quite agree. I started to get poorly with a whole heap of other stuff – diabetes, blood pressure...Everybody and his dog wanted to see me for an appointment for this, an appointment for that, and I’m like, ‘I’m still working!’ Taking time off to get a test for something I don’t have – well, what’s the point? If I had symptoms, I’d make an appointment. When I used to have smears, there was never anything abnormal anyway.  *[IN THEIR FIFTIES: Changing health concerns]*  **Eleanor:** I know what you mean with the health stuff, though. My rheumatism has changed everything. When I lie on that couch I can’t open my legs, it really hurts my joints...last time they had to pull me right down to the edge of the couch and I had to put my feet on the nurse. The laying down is ok, but putting my ankles together and opening my knees – [sharp intake of breath] ooh.  **Joanie:** Menopause, too – there’s enough to cope with!  **Eleanor:** Yeah, menopause. I felt completely dry down there – I couldn’t have sex, Bill and I were having real problems for a while. The nurse I saw said something really scarey, she made some kind of diagnosis or something, like is it atrophy? She didn’t explain. It frightened me. Saw the GP...she thought it wasn’t important, the sex issue. ‘Oh, if that’s all that’s bothering you...’. It was just so embarrassing. I was in for an appointment about something else when someone finally asked me what the problem was, I was coming out with stuff that I’d kept locked inside for years, really. That GP put me on oestrogen cream, I think it was – I had a few appointments to try this stuff out. It helped everything – made screening much less uncomfortable too – it’s still undignified, but you just have to get on with it, really, don’t you?  **Joanie:** In all the years I’ve missed screening, no-one’s ever asked me why, even when the these little overdue messages kept popping up on their screen when I went for other things – they just said ‘Book an appointment on your way out’, and I’d go ‘Yep’. Had a moment of guilt a few years though, I did try to book, but the appointment was weeks away – on the day I felt rubbish, I couldn’t go, I felt so guilty. It’s better not to make an appointment in the first place than to mess them around.  *[IN THEIR SIXTIES: Sex and relationship issues are changing for this generation]*  [laughter]  **Eleanor:**  I’ve only slept with Bill, and sex isn’t on the agenda much any more! I prefer gardening! I don’t really think I’m at risk, but you should get screened anyway, even if you have only slept with one person. You should go, Joanie!  **Joanie:** I know. I’m a young sixty – I’m still having a sex life! I’ve got other friends my age who are dating again. I was gonna moan about this actually – I’ve just found out I won’t get any more screening after I’m 64...I’m being pushed on the scrap heap, they don’t wanna know...not everyone’s past their sell-by date! And I need to make sure I go for one last check – otherwise it’s gonna to be too late. | 3: Developing rapport, considering past experiences.  3: History-taking, how feelings change over time dependent on experiences.  2. Matching patient and practice nurse, developing rapport.  4. Chronic health issues.  4. Addressing mobility issues.  3. Linking screening with sex and relationships.  3. Developing rapport, addressing difficulties..  2. Offering  pre-screening appointments.  3. Asking why screening is difficult.  3. Linking screening with sex and relationships. |
| **NARRATOR (WOMEN’S HEALTH EXPERT): CALL TO ACTION**  Thank you, Eleanor and Joanie.  What can Eleanor and Joanie’s stories tell us about the needs of women over fifty when we ask them to come for cervical screening?  We can see several things that have affected their willingness to attend, including the memory of what screening was like in their twenties and thirties, how lifestyles have changed over the years, and how aging can bring new concerns about changes in body image or new health problems which might make screening more difficult.  The research project I mentioned at the beginning of this video took women’s descriptions of their experiences to health care professionals, to look at what aspects of practice might make a difference. The project produced a call to action based on three key areas of good practice relating to women over fifty. These are:  First of all, be prepared for screening difficulties in this cohort of women:   - Get to know your patients. Who is at risk? How might you find out their concerns and address them? - Get to know your fellow screen-takers – network with experienced screening colleagues and colposcopists, who have seen these issues before and will be able to give you hints and tips about adapting procedures. Having an experienced, trained colleague available to advise during screening appointments is a second line of defence against problems.   Secondly, listen to your patient and address their individual concerns:   - All women have a history. Establish relationships with patients which take the context of their lives into account. Don’t underestimate the importance of finding out what their experiences of intimate examinations have been like in the past: these experiences are very diverse, and very individual. - Identify points of difficulty – take a step back before screening and address misunderstandings about risk or screening procedures. Do you need to take active steps to address anxiety, or physical problems linked to the menopause such as vaginal atrophy? - If your patient is a non-attender, take the step of asking ‘Why?’ a patient has decided not to attend.   Thirdly, consider a few adaptations for women over fifty, both in flexible appointment making, and in the practical procedures of screening. Think about:   - how appointment times fit with women’s needs (this will obviously also depend on the capacity of your practice) - who a woman might prefer to conduct her screening - whether repeat appointments are required to address difficult issues prior to screening   To adapt or tailor screening appointments to each individual, consider:   - finding out about ways of positioning women differently for their comfort, in particular in relation to mobility issues related to chronic illness; - getting to know how different sized speculums suit different women, - being clear with women who have been through menopause about where lubrication can be used on the speculum and why, and - helping women get to know their bodies – for example, the positioning of their cervix – this can then empower them to be more in control during subsequent intimate examinations.   So, to summarise, what’s the best way to approach cervical screening with women over fifty?  *[CALL TO ACTION]*  PREPARE: Get to know your patients, and get to know your cervical screening team;  LISTEN: Ask women about their past experiences and address any concerns; and...  ADAPT: If possible, offer women a choice about who carries out their screening and when, and consider alternative positions on the couch and lubrication and creams which might make screening easier for women in this age group.  Thank you for watching. | |
